# Health care access for rural youth on equal terms? A mixed methods study protocol in northern Sweden

**DOI:** 10.1186/s12939-018-0718-z

**Published:** 2018-01-11

**Authors:** Isabel Goicolea, Dean Carson, Miguel San Sebastian, Monica Christianson, Maria Wiklund, Anna-Karin Hurtig

**Affiliations:** 1 Unit of Epidemiology and Global Health, Department of Public Health and Clinical Medicine, Umeå University, Umeå, Sweden; 2 Demography and Growth Planning, Northern Institute, Charles Darwin University, Darwin, Australia; 3 Centre for Rural Medicine, Storuman, Sweden; 4 Arctic Centre at Umeå University, Umeå, Sweden; 5 Department of Nursing, Umeå University, Umeå, Sweden; 6 Unit of Physiotherapy, Department of Community Medicine and Rehabilitation, Umeå University, Umeå, Sweden

**Keywords:** Mixed methods, Rural, Youth, Health care, Access, Equity

## Abstract

**Background:**

The purpose of this paper is to propose a protocol for researching the impact of rural youth health service strategies on health care access. There has been no published comprehensive assessment of the effectiveness of youth health strategies in rural areas, and there is no clearly articulated model of how such assessments might be conducted. The protocol described here aims to gather information to; i) Assess rural youth access to health care according to their needs, ii) Identify and understand the strategies developed in rural areas to promote youth access to health care, and iii) Propose actions for further improvement. The protocol is described with particular reference to research being undertaken in the four northernmost counties of Sweden, which contain a widely dispersed and diverse youth population.

**Methods:**

The protocol proposes qualitative and quantitative methodologies sequentially in four phases. First, to map youth access to health care according to their health care needs, including assessing horizontal equity (equal use of health care for equivalent health needs,) and vertical equity (people with greater health needs should receive more health care than those with lesser needs). Second, a multiple case study design investigates strategies developed across the region (youth clinics, internet applications, public health programs) to improve youth access to health care. Third, qualitative comparative analysis of the 24 rural municipalities in the region identifies the best combination of conditions leading to high youth access to health care. Fourth, a concept mapping study involving rural stakeholders, care providers and youth provides recommended actions to improve rural youth access to health care.

**Discussion:**

The implementation of this research protocol will contribute to 1) generating knowledge that could contribute to strengthening rural youth access to health care, as well as to 2) advancing the application of mixed methods to explore access to health care.

## Background

This research protocol will lay the foundation for strengthening youth health care services in rural areas of northern Sweden by mapping youth access to health care services and critically evaluating strategies used to enhance access. The protocol emerges from both Swedish and international concerns about health status and health care access of rural youth [1, 2]. While it is well known that access to health care services continues to be a challenge for populations living in rural areas in Sweden (and elsewhere), existing research has focused mainly on the general population or on elderly groups, overlooking the situation of youth.

### Access to health care and young people

Despite youth health having generally improved in Europe in the last decade [3] youth remains a period when individuals face greater risk of morbidity and mortality associated with violence, mental health, and reproductive health problems [4]. Social inequalities in youth health persist and have even increased in certain European countries [5].

Young people’s access to health care services and preventive strategies are crucial to capture problems at an early stage. Health care services can have a beneficial impact on youth health as long as young people access them [4, 6–8]. Poor access to health care services for young people may result in both short-term harm and long-term disengagement with the services, causing poorer population health outcomes and higher overall costs to the health care system [9].

**Fig. 1:**
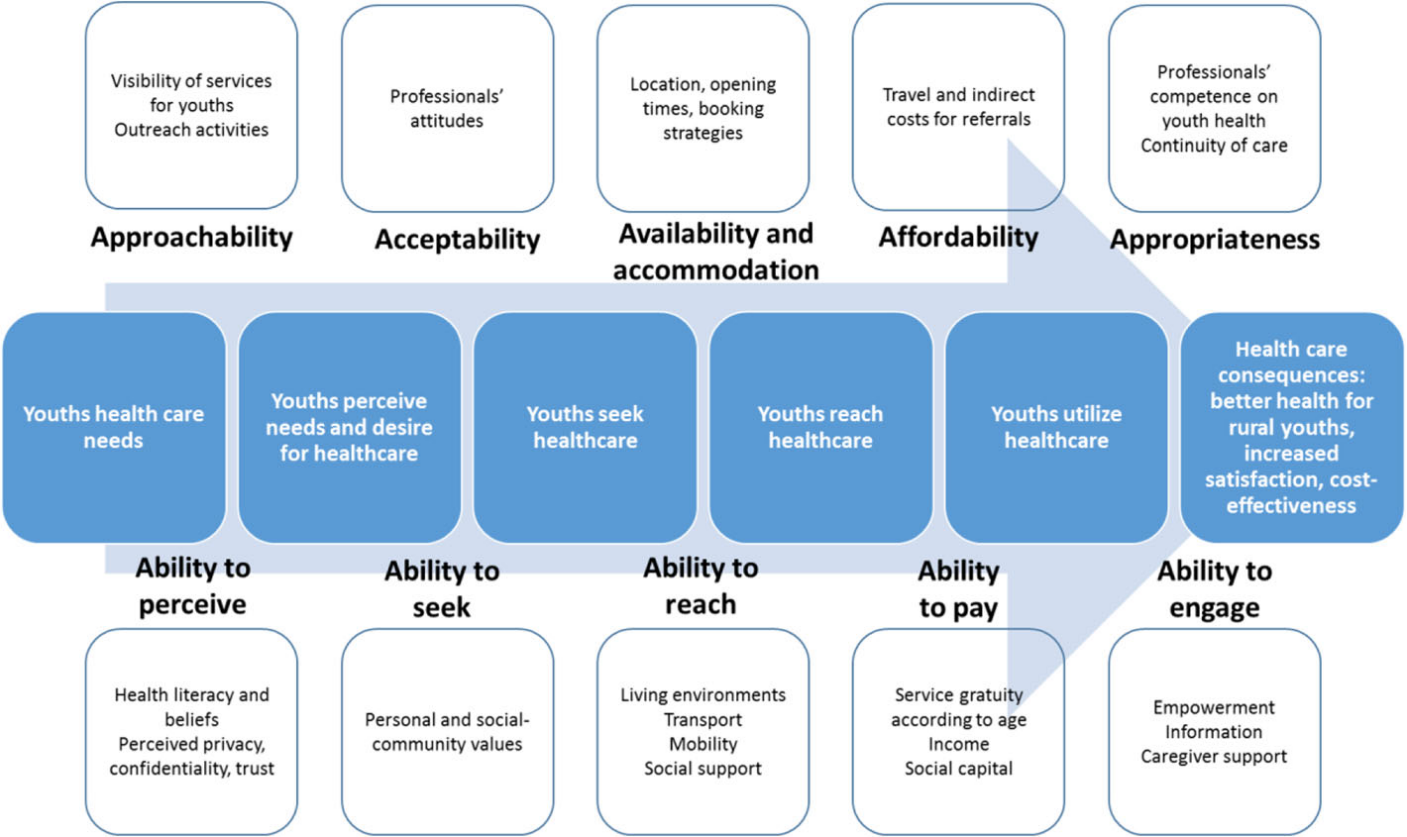
Conceptual framework to analyse access to health care for rural youth. Adapted from Levesque et al’s framework of patient-centred access to health care [10]

This protocol adapts Levesque et al.’s framework of patient-centred access to health care [10]; where access is defined as the possibility to identify healthcare needs, to seek healthcare services, to reach healthcare resources, to use healthcare services and to actually be offered services appropriate to the need for care. The framework points out five dimensions of access, capturing both supply and demand. On the supply side service accessibility is dependent on the dimensions of approachability, acceptability, availability, affordability and accommodation. On the demand side five abilities of the potential users influence their access: ability to perceive, to seek, to reach, to pay and to engage [10] (Fig. 1).

There are parallels between this framework and the World Health Organization’s (WHO) concept of youth-friendly health care services. According to WHO, for health care services to be accessed by youth they should be: accessible, acceptable, equitable, appropriate and effective for different youth subpopulations [4, 11]. More recently, WHO has proposed that such an approach should be fully integrated and sustained, aiming for youth-responsive health systems [12]. Some possible strategies to enhance youth access to healthcare include a) strengthening school health services, b) embracing e-health (healthcare practice supported by electronic processes and communication) and m-health (practice of medicine and public health supported by mobile devices) technologies, and c) undertaking community-based initiatives.

### Rural youth and (equal) health care access

Studies exploring access to health care for rural youth are rare in Sweden, with the exception of some consideration of (rural and urban) Sámi (the indigenous population in the northern part of Scandinavia and the Kola Peninsula) youth health status, but not of access to health care [13]. Studies in the US evidence the gap between the health needs of rural youth and the services they access. Rural youth experience poorer access to health care services – confidentiality concerns are amplified in rural settings and access to specialty care is limited [1]. A qualitative study in Australia identified lack of anonymity, a rural culture of self-reliance and stigma as barriers to seeking health care for mental health problems among rural youth, and highlighted that solutions designed for adults do not always work for young people [14] . Another study exploring access to sexual and reproductive health care services in rural Australia also found that while structural issues such as transport, cost and opening hours were important for young people, they placed greater value on personal attributes of service providers, particularly welcoming and non-judgmental attitudes [15].

The diversity aspect is important to highlight since rural youth are not a homogeneous group. An important number of northern Swedish rural youth are Sámi and the number of international migrants is increasing [2, 13]. In rural areas, as elsewhere, access to health care for youth might also be modulated by other axes of inequity based on gender, sexual identity/orientation, socio-economic status, education or ethnicity. For example, young men, those with trans experiences, from non-Swedish ethnic background and from lower socioeconomic status might face more barriers for accessing health care services [11].

In Sweden, there are a number of health care services where young people can access health information and/or care (i.e. health centres, student health, specialized mental health services/psychiatry, etc.) with youth clinics being the best known. There are 265 Youth Clinics in Sweden serving people ages 13–23. For accessibility and privacy, youth clinics are usually located away from general health services, and consultations are free of charge. Staffing and the menu of services vary between large and small youth clinics: the minimum team consists of a midwife and a social counsellor or psychologist, with a physician working some days [16].

Swedish youth clinics constitute one of the best examples of nationwide youth-friendly health care services [11]. However, youth clinics are not equally distributed across Sweden, and a large number of rural areas lack a youth clinic. Whether lack of access to youth clinics affects rural youth access to the health system more generally, and how rural municipalities that do not have youth clinics manage youth health care services, is not known.

This study aims to; i) Assess rural youth access to health care according to their needs, ii) Identify and understand the strategies developed in rural areas to promote youth access to health care, and iii) Propose actions for further improvement. The project focuses on northern Sweden as an example of a rural landscape, mixing qualitative and quantitative methodologies sequentially in four phases. The overall research question guiding the study is ‘What are the characteristics of rural municipalities and rural living which impact on youth access to health care?’.

## Methods/design

### Study setting

For this project, we will focus on the four northern counties of Sweden; which encompass 42 municipalities, 60% of the Swedish land area and only 12% of the population. Fifty seven percent (24 out of 42) of the municipalities are classified as rural by the Swedish Association of Local Authorities and Regions. The rural population of northern Sweden has been described as being older, with less education and more sedentary lifestyles than their urban counterparts [17]. However, young people aged 15 to 24 living in these counties account for around 11% of the population.

### Overall study design: Mixed methods research

This study aims to fill the identified knowledge gap by applying mixed methods research in four iterative phases (Table 1). In the first phase the dimension of horizontal and vertical equity in health care for youth in Northern Sweden will be assessed. In the second phase, through a multiple case-study we will unearth the strategies used by rural municipalities to enhance rural youth access to health care. In the third phase we will use qualitative comparative analysis to identify the key combination of conditions needed to achieve good access. Finally in the fourth phase, through a concept mapping study, a road map to improve rural youth access to health care services will be developed (Table 1).

**Table 1:** Phases, research questions and study designs

Phases	Research question	Study design	Sample size and approach	Data collection methods
1	Do young people living in rural municipalities have access to health care according to their needs?	Population-based cross sectional study.`	Probabilistic 3016 young people in 42 municipalities	Survey
2	What are the strategies implemented to strength rural youth access to health care?	Multiple case study	Purposive 6 rural municipalities	Interviews Observation Focus group discussions
3	What combination of conditions are key to ensure rural youth access to health care?	Qualitative comparative analysis using fuzzy sets	Purposive 24 rural municipalities	Interviews
4	What actions will improve rural youth access to health care?	Concept mapping	Purposive 80 stakeholders and young people	Brainstorming Free listing Sorting

Following a mixed methods approach, we have defined specific research questions to be answered in each of the phases with different methods, as well as an overall research question to be answered through integration of all the methods [18]. For the sampling, we will use stratified purposive sampling, meaning that we will start stratifying the larger group of interest (42 municipalities) in order to purposively select a smaller number of cases (6 municipalities), then move back to a larger number of cases (24 municipalities) and finally shift the focus from municipalities to stakeholders (80 stakeholders and youths). For the analysis, we will follow an iterative analysis involving sequenced phases where the conduct of each phase draws on the analysis of the preceding phase [18]. While phase 1 and 2 will involve quantitative and qualitative data collection and analysis respectively, phases 3 (qualitative comparative analysis) and 4 (concept mapping) constitute good examples of methodologies that go one step beyond in the qualitative-quantitative integration by supporting the notion that qualitative information can be well represented quantitatively and that quantitative information rests upon qualitative judgement [19].

The rational underlying mixed methods research is that the integration of qualitative and quantitative approaches allows the analysis of phenomena from different perspectives and provides an enriched understanding. Some authors also claim that mixed methods – and specifically concept mapping- can be used as transformative tools to contribute to positive social change [20]. In this research study, we consider that the last phase (concept mapping [21]) aims for transformation, namely proposing strategies for change and improvement of health care access for rural youth.

### Phase 1. Mapping rural youth access to health care and inequities analyzing the “health on equal terms” survey

In the first phase we will map the current situation of youth access to health care, establishing comparisons between rural and urban areas, as well as analyzing the role played by youth clinics. Socio-economic and gender inequities in health care utilization will also be examined. We will analyze secondary data from the most recent ‘Health on equal terms’ survey conducted in the four northern counties in 2014 [22].

This survey is conducted by the respective county councils, and represents a regionally expanded sample (*N* = 50,300 invited, 50% response rate) of the annual national HET survey managed by the National Public Health Agency of Sweden, in collaboration with the county councils and Statistics Sweden (SCB). HET in northern Sweden is based on a population random sample stratified into 276 strata by county, municipality, gender and age. The total number of youth in the sample of the four northern counties is 3016. HET is conducted for policy-making monitoring purposes and to our knowledge no power calculations have been carried out.Equity of access to health care is hard to measure [23]. Since equal access can rarely be observed directly, what is commonly measured and analysed is equal utilization, although there are limitations with this approach as it does not account for acceptable variations in the use of healthcare [24]. In this study, access to health care will be operationalized through self-reported utilization of health care services.

We will use the horizontal inequity index (HII) to operationalize horizontal equity [25, 26]. HII is defined as the difference between observed health care utilization (actual use of the service) and that which would be expected given the individual’s health needs (more health needs, more use). Once health needs are standardized across individuals, the remaining utilization can be attributed to non-need factors and therefore considered to be inequitable. The HII will be calculated using: i) health care need factors (i.e. sex, age, self-reported health and previous medical conditions); ii) non-need factors (education, income, municipality of residence); and iii) health care utilization information in the last year (visit to a youth clinic, visit to a general practitioner, visit to a midwife, among others). This analysis will be complemented by regression models with health care utilization as the outcome variable, the non-need factors as the exposure and adjusting for health care need factors.

For vertical (in)equity, two kind of variables will be used: i) health need defined as the severity of one or several health outcomes (like self-reported health or previous medical conditions) and ii) health care utilization as previously defined. Regression models will be developed including health care utilization as the outcome variable and health need as the exposure adjusting for different socioeconomic factors.

HII and regression models will be estimated and comparisons with the youth population in urban areas will be made, also disaggregated by gender. Comparisons between rural municipalities with and without a youth clinic will also be conducted.

### Phase 2. Understanding strategies to improve rural youth access to health care through a multiple-case study

The previous analysis will allow us to stratify the 42 municipalities in terms of rurality, vertical and horizontal equity in access and the presence or not of a youth clinic. On this basis we will purposively choose six rural municipalities (three representing an equal access (HII close to zero) and three an unequal access; two of them with a youth clinic and four without a youth clinic) to explore the strategies they employ to ensure youth access to health care services that is responsive to their diverse needs. We will examine how issues of gender, ethnicity and sexuality are addressed using this multiple case study design [27]. Strategies might include the use of internet and other technologies, coordination with nearby youth clinics, and development of youth oriented public health programs, to cite a few.

From each case (rural municipality) qualitative data will be gathered through observation, document review, interviews and focus group discussions. Collection of multiple sources of data is a requirement and a strength of the exploratory case studies, since it allows triangulation across data sources [27].

Non –participant observation will be conducted in three selected settings where young people seek health care -health care centres/community hospitals, school-health and youth clinics. The objective of the observations will be to familiarize the researchers with the setting and to gather information regarding the organizational context and interaction with young people. Documents will also be collected including plans, projects, guidelines, and evaluations relevant to youth and health care.

Semi-structured interviews will be conducted with different stakeholders involved with youth and health, such as social workers, school nurses, teachers, and health professionals working in primary health care, mental health and youth clinics. Approximately 5–10 interviews will be conducted in each of the six cases. In addition, focus group discussions will be held with young people. Since youth experiences might differ based on their gender and ethnicity, we will make sure that participants in the groups reflect the diversity of the youth population in each case - i.e. different genders, ethnic backgrounds. Approximately 3–5 focus group discussions will be carried out in each of the six cases. Stakeholders will be recruited through already existing contacts following snow-ball sampling. For the focus groups discussions, young people will be recruited through schools and in municipalities with youth clinics, via the clinic. In order to capture the perspectives of youth sub-populations that might be in more vulnerable situations (LBGTQI+ youth, immigrant youth and Sami youth), alternative ways to recruiting youth from these subgroups will also be used - i.e. through Sami organizations, civil society organizations working with immigrant youth and LBGTQI+ networks. In small municipalities, where focus group discussions with youth from such subgroups will not be feasible or safe to conduct, individual interviews will be conducted.

Guides for the individual interviews and focus group discussions will be developed in order to explore issues such as: perceptions about youth access to health care according to their needs (individual and community influences as well as health care services characteristics), strategies developed to enhance access, perceptions of the different health care services available, referrals, coordination between different sectors, perceptions on inequities in access for different youth subgroups and strategies used to minimize such inequities. All the material will be audio recorded and transcribed verbatim.

The data analysis will broadly consist of two phases. First, we will set out to develop thick descriptions of each case [8, 27]. We will use a deductive thematic analysis approach [28] whereby we will start with predefined codes derived from the conceptual framework described in Fig. 1. Those concepts will serve as the main guides to analyze the interview transcripts (that will be verbatim transcribed and imported into Open Code 4.03 for data management and coding), as well as the notes taken during observation. However, we will remain open to other relevant issues not predefined. Codes will be aggregated until the final themes emerge. The second stage will focus on establishing comparisons across cases looking for both similarities, differences and patterns in order to identify conditions (factors) that participants consider key to ensure good youth access to health care [27].

### Phase 3. Identifying best practices using qualitative comparative analysis

The conditions emerging from the multiple case study as important for ensuring good health care access for young people will be further assessed in all the 24 rural municipalities. We will focus on conditions belonging to the health care services domains –approachability, acceptability, availability-accommodation, affordability, and appropriateness-, since those are the ones that can be most readily modified in the short-run [10]. We cannot determine beforehand exactly which those conditions will be, since they will emerge from the analysis of the cases. Based on existing literature, conditions that have been found to be important for ensuring youth access to health care within each of these domains are described in Table 2.

**Table 2:** Five dimensions of accessibility of services and potential conditions to be assessed (to be further refined in the light of the analysis conducted in Phase 2)

Domains	Potential conditions
Approachability	• Visibility of health care services for youth • Outreach activities • Existence of youth clinic
Acceptability	• Health care professionals’ attitudes towards youth: motivation, interest
Availability and accommodation	• Health care services location and array of services provided • Opening hours • Appointment systems • e-health strategies
Affordability	• Gratuity of services • Travel arrangements to reach services
Appropriateness	• Health care professionals´ knowledge and skills in youth health: training received, knowledge, self-efficacy • Coordination and referral networks • Policies and guidelines for youth health

For each condition, indicators will be developed and information gathered using secondary data and structured interviews with representatives of each of the municipalities. For the outcome we will use the horizontal equity index calculated in Phase 1. The connection between conditions and outcome will be analysed using qualitative comparative analysis (QCA) with fuzzy sets [29].

Qualitative comparative analysis (QCA) studies cases as configurations of conditions. It allows the combination of both a sufficiently deep exploration of individual cases and the identification of patterns across-cases, namely combinations of causal conditions that are connected with different outcomes. QCA uses Boolean algebra to assess the extent to which a configuration of conditions explains outcomes, in terms of necessity - whether the cause is present in all (or almost all) the instances of the outcome-, and sufficiency - namely whether the cause is invariably (or almost) followed by the outcome. The underlying principles are that: i) a condition causes an outcome not in isolation but in combination with other conditions (conjunctural causation), and ii) there are more than one of those causal combinations leading to the same outcome (equifinal causation). The final step of the analysis consists of the development of a solution formula that presents the combination of conditions that best explain the outcome [29, 30]. In this study QCA analysis will be used in order to identify the best combination of conditions to ensure rural youth access to health care.

### Phase 4. Proposing a way forward through a participatory concept mapping study

Phase 4 will consist of a participatory process, in which stakeholders and young people from the local, regional and national levels will be involved in developing a Road map containing key strategies to improve rural youth access according to their diverse needs. First, stakeholders and youth who we had identified through the implementation of this research project will be invited to a workshop where the preliminary results will be presented and distributed via a short summary. Using these results as a basis to reflect upon, participants will be asked to participate in a concept mapping study to collectively develop a set of actions to strengthen rural youth access to health care.

Concept mapping enables groups of actors to visualize their ideas around an issue of mutual interest and develop common frameworks through a structured, participatory process. Qualitative and quantitative data are generated and integrated by participants through sequential steps, beginning with generation of ideas (brainstorming), structuring of ideas through sorting and rating, development of conceptual maps based in multivariate statistical methods, and finally collective interpretation of the maps [21].

A focus question to orient the brainstorming activity will be developed by the research team and sent via email, together with clear instructions to all of the participants in the first workshop and other relevant stakeholders and youths. For participants that would might not want to use the electronic version, a phone or face to face interview will be arranged.

The focus question will aim to identify strategies/actions that should be implemented to strengthen youth access to health care services in rural areas. The actual phrasing of the focus questions will build upon the findings from the previous stages of this study.

The research team will gather all the proposed actions and refine the list by deleting duplicates, merging or splitting proposed actions. The refined list of actions will be sent again to a number of participants for sorting and rating. For the sorting, participants will be asked to group together actions in as many groups as they wish. For rating, the participants will be asked to rate the actions in terms of both relevance and feasibility. We will make sure to get equal participation in the concept mapping from both youth and other stakeholders, so that we can develop at least two different maps (one with the strategies prioritized by the youth and the other with the strategies prioritized by others) to be used as a starting point for the discussions in the workshops.

The gathered data will be analysed using concept mapping techniques that facilitate visualization of thematic clusters, and identification of areas of consensus for action. The Concept System- CS Global Max software will be used during the entire process.

Finally, all the qualitative and quantitative information will be integrated in order to answer our main research question. A Road Map with the main actions identified for improving rural youth access to health care in Sweden will be produced and presented in a final workshop with young people and relevant stakeholders.

### Ethical considerations

Clearance has been granted from Umeå Regional ethical review board (Dnr. 2017–217-31). The CIOMS guidelines will be followed as well as other ethical aspects specifically related to research with young people [31]. When young people participate in research projects, ethical considerations have to be taken into account in order to both respect their autonomy to make decisions and safeguard their wellbeing. Only participants aged older than 15 will be invited to participate which means that they can give autonomous consent, and efforts will be made to ensure that they understand what their participation involves. Written voluntary informed consent will be sought from each of the informants. Confidentiality and privacy will be guaranteed. The participants that will be included in this research project are not patients and the risk for dependency is therefore low. When publishing the information, pseudonyms will be used both for the participants and the municipalities and any information that might allow identification will be removed.

For the use of secondary data, relevant permissions will be obtained to access anonymized information. For the interviews with stakeholders and focus group discussions with young people, a private place will be located, and voluntary informed verbal and written consent will be sought. In the focus groups discussions we cannot guarantee true anonymity for the participants, but they will be assured confidentiality. Information gathered will be carefully protected. The unit of the analysis will be the group, not the individual. Participants in the focus group discussions will be informed about their obligation to respect the privacy of other focus group members by not disclosing personal information that they learn of during the discussions. For the interviews with municipal representatives, informed consent will be sought beforehand. Before the concept mapping study all potential participants will be invited to a workshop where they will receive further information, including ethical issues. Informed consent will be sought from each informant.

## Discussion

In this paper we describe a protocol that uses mixed-methods to analyse rural youth access to health care according to their needs, and propose actions for further improvement. The implementation of this research protocol will contribute to 1) generating knowledge that could contribute to strengthening rural youth access to health care, as well as to 2) advancing the application of mixed methods to explore access to health care.

The starting point of this proposal is an identified knowledge gap in the fields of rural health, youth health and access to health care. While research has pointed out that health care services are, in general, less accessible for young people [4, 9], few such studies have focused on rural youths. Additionally, research has also pointed out that rural populations face, in general, greater challenges to access health care services [32, 33] but such studies have focused on the general population and the elderly, overlooking youths. Findings from these two lines of inquiry give us a basis to hypothesize that rural youth access to health care might be inadequate given their needs and that strategies for improving access might be needed. Even more, not all rural youth subpopulations might be equally affected. However, there is very limited research to actually support this conjecture, especially when it comes to the Swedish context. Research aiming at identifying creative strategies designed in rural areas to cope with access challenges is also scarce, even if such strategies should play a key role when designing policies and actions for rural development and health.

This proposal can also contribute to advancements in the field of youth-friendly health care services. Research exploring the implementation of youth-friendly health care services have focused on urban areas and hospitals or specialized/specific services, i.e. mental health, contraceptive services [4, 6]. This disregards that an important number of young people live in rural areas, with less access to differentiated and specialized services. Generalizations made from studies in urban areas might not be applicable to rural settings, since the strategies to improve access to health care services for rural youth might be different from those used in urban areas.

This proposal can also contribute to the field of equity in access to health care, by empirically applying a framework that conceptualizes access as the interface between individual-community abilities and the accessibility of providers, organisations, institutions and systems [10]. Rural youth access to health care will be assessed using multiple methods in order to best capture the complexity of the concept, focusing on aspects of equity. There is a scarcity in the literature of studies exploring access integrating different perspectives and domains, and even less focusing specifically on rural youth, where besides the generic individual abilities and health system characteristics, other youth/rural specificities might play a key role in shaping access.

This proposal is grounded in the principle that all population groups should have access to health care services according to their needs, both as a basic human right and as a means for improving the health of the population [33]. The starting point is the assumption that health care needs are diverse for different population subgroups and that access is not equally distributed. The first axis of inequity that is in focus in this proposal is age/generation: health care services are less accessible for young people and less responsive to their needs [4, 9]. The second axis in focus is rurality: rural populations face greater challenges to access health care services due to the characteristics of rural communities (e.g. distance, harder to ensure privacy, culture of self-reliance) and health care services’ factors (e.g. harder to recruit and retain professionals) [32, 34].

In addition, we also plan to consider 1) how gender, sexual and ethnic-cultural diversity are perceived to influence access, and 2) how such factors are considered in the design and implementation of strategies to improve youth access to health care services. The diversity aspect is important to highlight since rural youth are not a homogeneous group [35]. In rural areas, as elsewhere, access to health care for youth might also be modulated by other axes of inequity based on gender, sexual identity/orientation, socio-economic status, education or ethnicity [2]. Studies in Australia and Norway, for example, have shown how culture-specific factors influenced indigenous youth utilisation of health care [14, 36].

Methodologically, this proposal also seeks to contribute to further development of mixed methods approaches for health care research. The complexity of the issues explored in this research area and the need for a range of methodologies to understand and evaluate these complexities has been pointed out as a rationale for promoting the use of mixed methods in health care research in general and in health care access research specifically [10]. Despite the potential advantage of mixed methods research in analysing and understanding complex health care systems issues, mixed methods as a methodology is still developing, and a number of debates remain open around philosophical assumptions, definitions, integration in research questions, data collection, data analysis, and interpretation [18]. While a large amount of mixed methods research claims to be grounded on pragmatism, this research project aligns with critical realism in the sense that quantitative analysis will be employed to identify patterns and correlations and qualitative analysis to trace causal mechanisms to better understand rural youth access to health care. Such an approach to mixed methods research has been used mainly in political sciences, but is less widespread in health care research, despite its potential to advance research in this discipline [37, 38].

Good access to health care for all (youth) populations, regardless of geography remains a key goal of governments and societies internationally [33]. The European Rural Manifesto adopted in 2015 asserted the right of the 150 million people living in rural areas to a quality of life equal to that of urban populations and called for actions to reverse the decline in rural areas [39]. Within rural development policies and plans, rural youth health and access to health care have not received much attention, which could be a consequence of the scarcity of research on this topic. There is an urgent need to extend the currently limited research to ensure that health services in rural areas provide the best environments for the youth that they serve. Looking globally, there are several areas that are geographically and demographically similar to the Swedish rural context, such as Canada, Australia, Norway, Finland and Scotland [40], where findings emerging from this study will be relevant.
